# Novel *GALC* Mutations Cause Adult-Onset Krabbe Disease With Myelopathy in Two Chinese Families: Case Reports and Literature Review

**DOI:** 10.3389/fneur.2020.00830

**Published:** 2020-08-21

**Authors:** Junfei Zhong, Fei Jiang, Huan Yang, Jing Li, Jian Cheng, Qiuming Zeng, Qian Xu

**Affiliations:** ^1^Department of Neurology, Xiangya Hospital, Central South University, Changsha, China; ^2^Department of Neurology, The Third Affiliated Hospital of Hunan University of Chinese Medicine, Zhuzhou, China; ^3^National Clinical Research Center for Geriatric Disorders, Xiangya Hospital, Changsha, China; ^4^Key Laboratory of Hunan Province in Neurodegenerative Disorders, Central South University, Changsha, China

**Keywords:** Krabbe disease, adult onset, novel *GALC* mutations, myelopathy, literature review

## Abstract

Krabbe disease (KD), also referred to as globoid cell leukodystrophy, is a rare autosomal recessive lysosomal storage disorder caused by β-galactocerebrosidase (GALC) deficiency. Most patients affected by this disease are infants, and <10% of cases suffer from adult-onset KD. In this study, two Chinese males presented with long-term progressive weakness in their limbs. Magnetic resonance imaging of the brain and spinal cord of these patients revealed lesions with abnormally high signal intensity on T2-weighted (T2W) and T2W fluid-attenuated inversion recovery images. Whole-exome sequencing was performed for both patients, and four *GALC* mutations were identified. Case 1 carried a novel deletion mutation (p.T633Tfs^*^2) and a known missense mutation (p.T529M), while case 2 carried a novel missense mutation (p.W355C) and a known missense mutation (p.P154H). Previous literature has rarely reported myelopathy in patients with KD; in this study, we report two cases of adult-onset KD who both experienced myelopathy. We also conducted a literature review of KD and its association with myelopathy. Our findings provide a better understanding of the phenotypic and genotypic profiles associated with adult-onset KD. We recommend that physicians consider KD as a possible diagnosis in cases showing progressive motor dysfunction or gait disorder in association with typical myelopathy.

## Introduction

Krabbe disease (KD), also referred to as globoid cell leukodystrophy, is an autosomal recessive lysosomal storage disorder caused by a deficiency of the lysosomal enzyme β-galactocerebrosidase (GALC). Mutations within the *GALC* gene lead to abnormalities in the GALC enzyme, resulting in the abnormal accumulation of its substrate psychosine in both the central and peripheral nervous systems. KD is characterized by multinuclear macrophages (globoid cells), extensive demyelination, and gliosis (1). The incidence of KD is ~1:100,000–1:250,000, and more than 90% of cases are diagnosed with the severe infantile-onset form of KD (2). The adult-onset form of KD is not fatal but results in high levels of disability; this form of KD has a milder, more variable phenotype and is associated with spastic paraparesis, ataxia, gait disorder, dysarthria, numbness, and so on. The genetic profile of KD is heterogeneous; over 128 mutations have been reported to cause KD, including missense, deletion, frameshift, non-sense, and insertion mutations (1). Herein, we describe two cases of adult-onset KD with myelopathy from two Chinese families. The patient in case 1 presented with tetraparesis, hypoesthesia, urinary and erectile dysfunction, and constipation. The physical examination revealed limbs weakness, muscle atrophy, the absence of bilateral abdominal and cremasteric reflexes, sensory loss at the T2 level, deep sensory impairment, tendon hyperreflexia, and bilateral positive Babinski signs. Magnetic resonance imaging (MRI) of the brain and spinal cord revealed lesions with abnormally high signal intensity on T2-weighted (T2W) fluid-attenuated inversion recovery (FLAIR) images. Whole-exome sequencing (WES) identified a novel deletion mutation (p.T633Tfs^*^2) and a known missense mutation (p.T529M). The patient in case 2 presented with tetraparesis, an unsteady gait, numbness, and mild dysarthria. The physical examination revealed limbs weakness, muscle atrophy, fasciculation, absent deep tendon reflexes, and bilateral positive Babinski signs. MRI of the brain and spinal cord also revealed lesions with abnormally high signal intensity on T2W and T2W FLAIR images. WES identified a novel missense mutation (p.W355C) and a known missense mutation (p.P154H). Myelopathy in patients with KD was rarely reported in previous literature; in this study, we report two cases of adult-onset KD who both experienced myelopathy, and present a review of the literature relating to KD and myelopathy.

## Case Description

### Case 1

**Figure 1:**
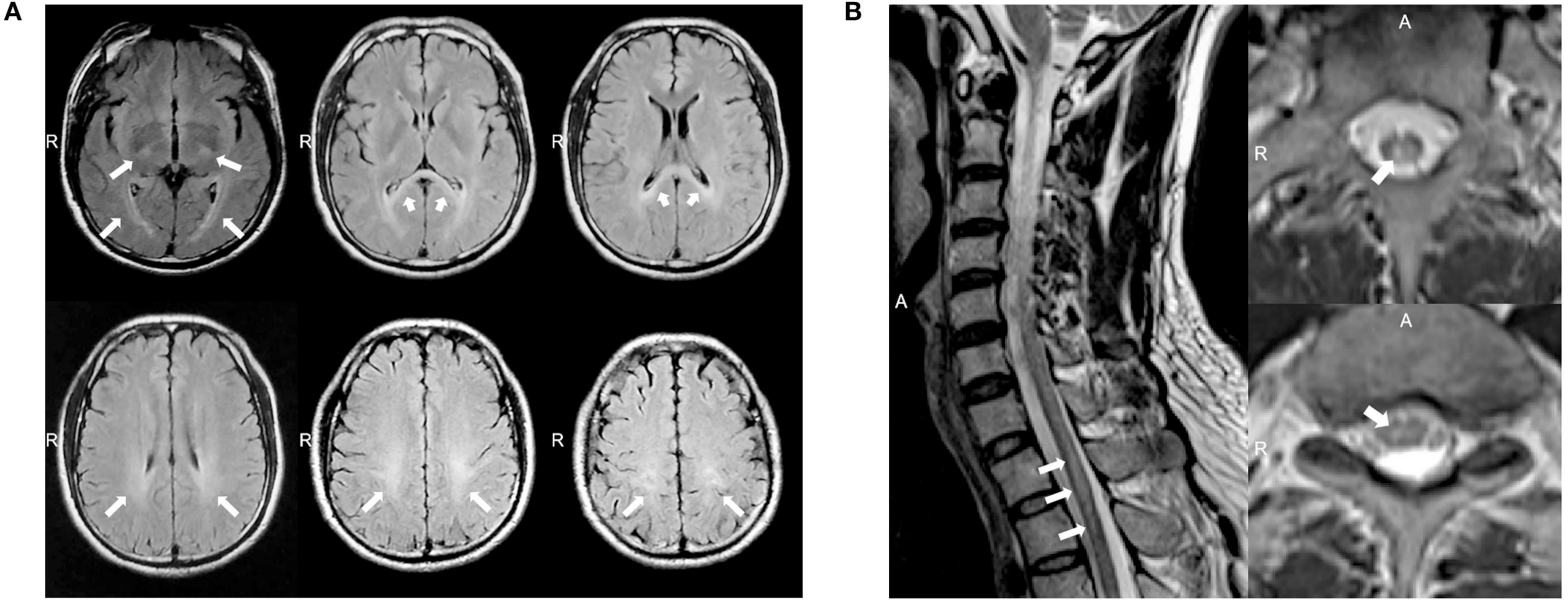
**(A)** Magnetic resonance imaging (MRI) of the brain for case 1 showed abnormal consecutive hyperintensities along the bilateral corticospinal tracts and in the optic radiation and corpus callosum on T2-weighted (T2W) fluid-attenuated inversion recovery (FLAIR) images (white arrowheads). **(B)** MRI of the spinal cord for case 1 showed abnormal intramedullary lesions from T1 to T2 on T2W images (white arrowheads).

A 37 year-old Chinese male presenting with weakness and numbness in the limbs was admitted to our neurology department. He had first presented clinical symptoms at 27 years of age, when he noticed a slight limp caused by weakness in his left leg. Over the next decade, he developed progressive weakness in his other limbs and gradually developed constipation, urinary and erectile dysfunction, and hypoesthesia, successively. When he was 37 years of age, the weakness in his lower limbs increased rapidly over a short period of time; this caused the patient to require a wheelchair. The patient received oral medications including mecobalamine, vitamin B1, gabapentin, and folic acid and rehabilitation training while little improvement was observed. On examination, the patient scored 4/5 and 2/5 in power tests for the upper and lower limbs, respectively. We also observed mild lower limb muscle atrophy, the absence of bilateral abdominal and cremasteric reflexes, tendon hyperreflexia, and bilateral positive Babinski signs. Sensitivity to a pinprick was diminished below the T2 level, and the patient had deep sensory impairment. The patient's score on the Barthel index was 35. Analysis of cerebrospinal fluid (CSF) revealed normal levels of protein (0.35 g/l), cell count, and glucose and negative oligoclonal bands. The values of serum cortisol and plasma adrenocorticotrophic hormone at 8 am were 8.34 μg/dl (normal range: 6.2–19.4 μg/dl) and 7.73 pmol/l (normal range: 1.6–13.9 pmol/l), respectively. The value of arylsulfatase A was 144.60 nmol/17 h/mg (normal range: >58 nmol/17 h/mg). Nerve conduction studies revealed axonal motor neuropathy (Supplementary Table 1). Visual evoked potential was normal. Somatosensory evoked potential indicated a delay in the spinal cord in the lower extremities. The needle electromyography (EMG) showed reduced motor unit numbers in the lower limbs, which indicated the neuropathy. MRI showed consecutive hyperintensity along bilateral corticospinal tracts and in the optic radiation and corpus callosum on T2W FLAIR images (Figure 1A). Notably, MRI of the spinal cord also revealed abnormal hyperintensities in the thoracic cord at T1–T2 on T2W images (Figure 1B).

**Figure 2:**
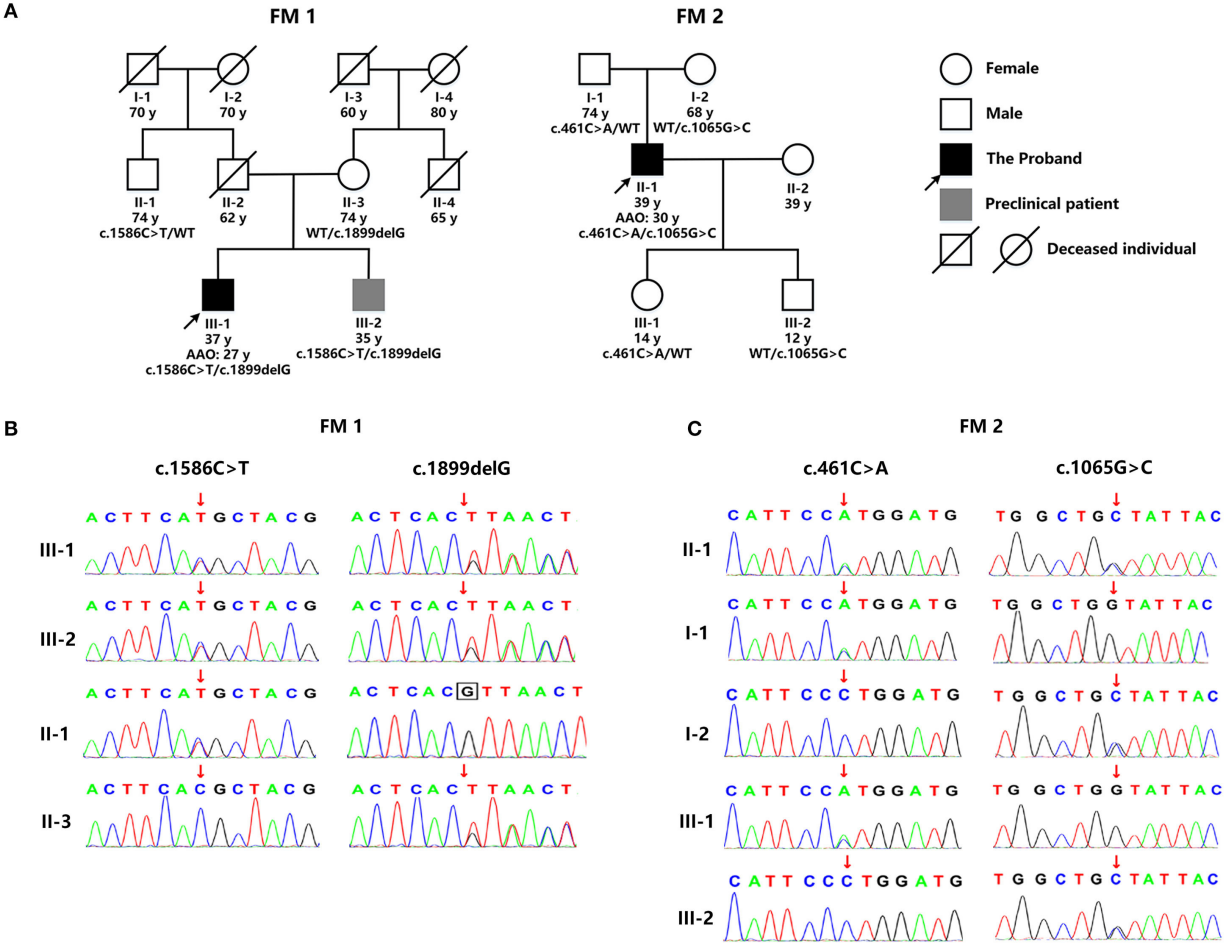
**(A)** The genetic pedigree for case 1 (FM 1) and case 2 (FM 2). **(B)** *GALC* gene sequencing of the family members of case 1 (FM 1) and case 2 (FM 2). FM, family; AAO, age at onset; WT, wild type.

WES detected two mutations in *GALC*: a novel deletion mutation [c.1899delG (p.T633Tfs^*^2)] and a known missense mutation [c.1586C>T (p.T529M)] (Figure 2B). p.T529M has been reported in KD patients previously (3). The novel mutation in exon 16, p.T633Tfs^*^2, was predicted to result in the premature termination of translation and was not listed in the dbSNP (version 153), 1,000 Genomes (Phase 3), and ExAC databases (version release 1). The enzymic activity of GALC in peripheral blood leukocytes from the patient had fallen to 1.04 nmol/17 h/mg (normal range: >18 nmol/17 h/mg), thus supporting the diagnosis of KD. There were no reports of consanguineous marriage within this family. Although the patient's 35 year-old brother was asymptomatic, clinical examination revealed Pes Cavus, tendon hyperreflexia, bilateral ankle clonus, and positive Babinski signs. Their father had died in an accident at 62 years of age, and their mother had a 19 year history of schizophrenia. The paternal uncle was normal (Figure 2A). With consent, we performed further genetic analysis on other members of the family. The younger brother of the proband carried the same compound heterozygous mutations (p.T633Tfs^*^2 and p.T529M), while their mother and the paternal uncle were heterozygous carriers of p.T633Tfs^*^2 and p.T529M, respectively (Figure 2B). Collectively, this information indicates that p.T633Tfs^*^2 is a pathogenic mutation and causes KD.

### Case 2

**Figure 3:**
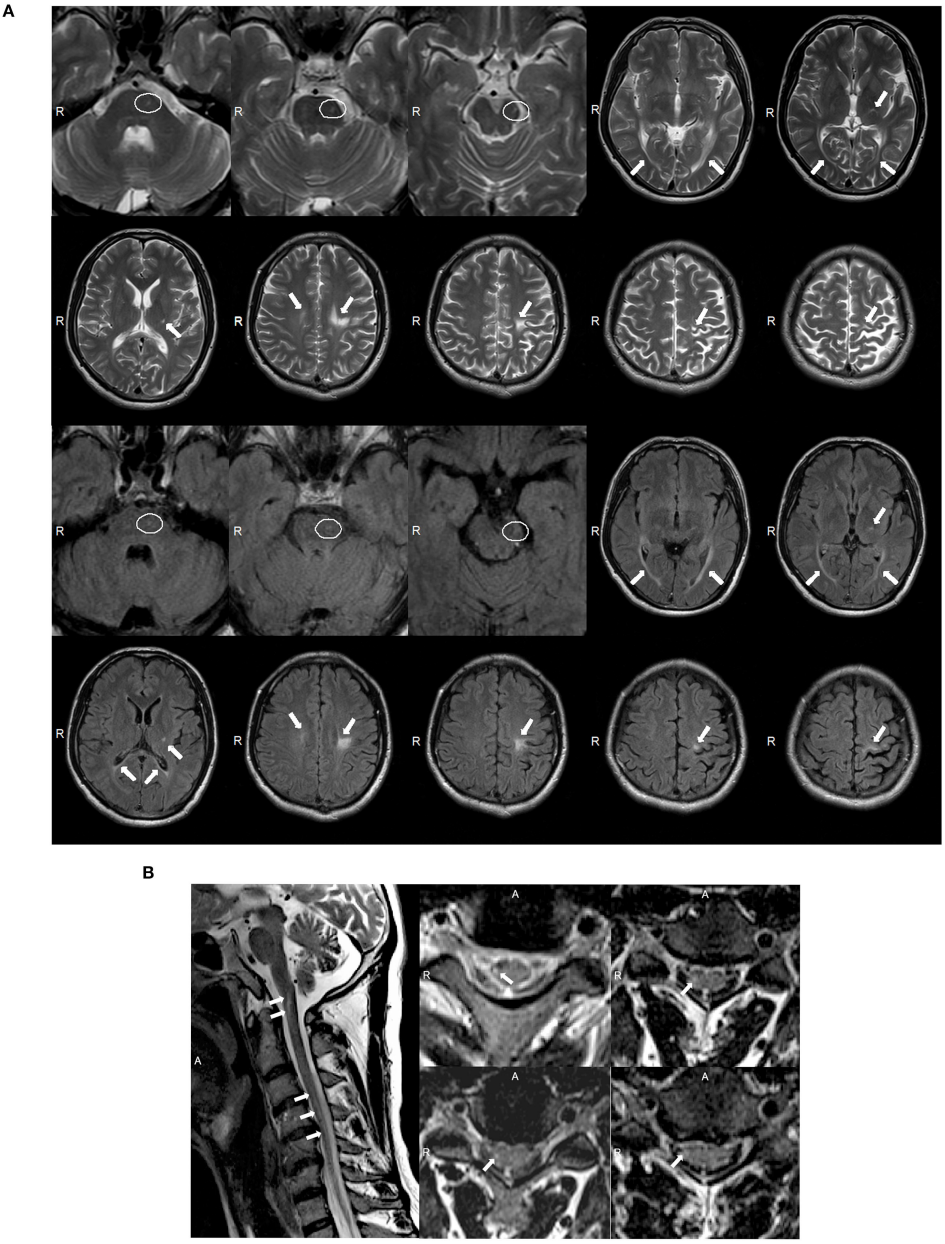
**(A)** Magnetic resonance imaging (MRI) of the brain for case 2 showed abnormal asymmetric consecutive hyperintensity along the bilateral corticospinal tracts (from the precentral gyrus down to the pons on the left and in the level of the centrum semiovale on the right), and in the optic radiation and corpus callosum on T2-weighted (T2W) and T2W fluid-attenuated inversion recovery (FLAIR) images (white arrowheads). MRI images of the mesocephalon have been enlarged to highlight the lesions on the pons (white ovals). **(B)** MRI of the spinal cord of case 2 showed abnormal inconsecutive intramedullary lesions from C1 to C5 on T2W images (white arrowheads).

A 39 year-old Chinese male was admitted into our neurology department because of a history of tetraparesis lasting 9 years. At 30 years of age, he had noticed that he could not hold objects stably with the right hand. Since then, he developed progressive weakness in the four limbs, an unsteady gait, numbness, and mild dysarthria. He successively received glucocorticoid pulse therapy, intravenous immunoglobulin, and plasma exchange; however, there was no improvement and his symptoms worsened. On examination, he scored 4/5 in power tests of the upper and lower proximal extremities and 3/5 in distal extremities. We observed atrophy of the bilateral interphalangeal muscle, the thenar and hypothenar muscles, and supraspinatus and infraspinatus; fasciculation; absent deep tendon reflexes; and bilateral positive Babinski signs. The patient's score on the Barthel index was 85. Analysis of CSF showed elevated protein levels of 0.69 g/l but normal cell counts and glucose levels. Oligoclonal bands were negative. The value of arylsulfatase A was 133.97 nmol/17 h/mg (normal range: >58 nmol/17 h/mg). Nerve conduction studies revealed severe motor and sensory demyelinating polyneuropathy associated with axonal damage (Supplementary Table 2). The needle EMG showed fibrillation potentials and positive sharp waves in the right biceps and tibialis anterior. Wide duration, high amplitude, and polyphasic form were observed in the right biceps during mild voluntary activity, which indicated neuropathy. MRI of the brain showed asymmetric consecutive hyperintensity along bilateral corticospinal tracts (from the precentral gyrus down to the pons on the left and in the level of the centrum semiovale on the right) and in the optic radiation and corpus callosum on T2W and T2W FLAIR images (Figure 3A). MRI of the spinal cord revealed inconsecutive lesions with high signal intensity in the cervical cord at C1–C5 on T2W images (Figure 3B).

WES detected two missense *GALC* mutations in this patient: c.461C>A (p.P154H) and c.1065G>C (p.W355C) (Figure 2B). p.P154H has been reported previously in KD patients (4). However, the p.W355C mutation in exon 10 was novel and predicted to be potentially pathogenic by a range of *in silico* prediction tools, including Mutation Taster, SIFT, Polyphen, and Provean. Furthermore, this mutation was not listed in the dbSNP (version 153), 1000 Genomes (Phase 3), and ExAC databases (version release 1). The patient refused to undertake tests for GALC enzyme activity. There were no reports of consanguineous marriage within this family, and all other family members were normal (Figure 2A). With consent, we performed genetic analysis for other members of the patient's family. The patient's father and daughter were both heterozygous for p.P154H, while his mother and son were both heterozygous for p.W355C (Figure 2B). Collectively, this information indicates that p.W355C is a pathogenic mutation and causes KD.

## Discussion

Herein, we report two Han Chinese males who were diagnosed with adult-onset KD with myelopathy. WES identified four *GALC* mutations. Case 1 carried a novel deletion mutation (p.T633Tfs^*^2) and a known missense mutation (p.T529M), while case 2 carried a novel missense mutation (p.W355C) and a known missense mutation (p.P154H). Case 1 presented with slow but progressive weakness in his limbs, numbness, and pyramidal signs such as tendon hyperreflexia and bilateral positive Babinski signs. These symptoms were consistent with the clinical presentations of most other patients with adult-onset KD reported in existing literature. However, case 1 also presented with less commonly reported symptoms, including constipation, urinary and erectile dysfunction, muscle atrophy, the absence of bilateral abdominal and cremasteric reflexes, sensory loss at the T2 level, and deep sensory impairment (5). MRI of the brain showed leukodystrophy typical of adult-onset KD patients; T2W FLAIR images revealed hyperintense lesions involving the corticospinal tracts, optic radiation, and corpus callosum (6). MRI of the spinal cord further revealed hyperintensity in the thoracic cord affecting the gray matter of the spinal cord and the posterior funiculus at the T1–T2 level on T2W images; these factors resulted in sensory loss at the T2 level, along with constipation, and both urinary and erectile dysfunction. The nerve conduction studies revealed axonal motor neuropathy, which contributed to the gait disturbance along with pyramidal tract dysfunction. WES identified two *GALC* mutations in this patient, and the diagnosis of KD was confirmed by reduced GALC enzyme activity. It is possible that myelopathy may have contributed to the deterioration of weakness in the lower limbs over a short space of time.

Case 2 also presented with slow but progressive weakness in the limbs, numbness, dysarthria, gait disorder, and positive Babinski signs. These are common symptoms in patients with adult-onset KD; rarer symptoms include muscle atrophy and fasciculation (5). While case 1 experienced tendon hyperreflexia, there was an absence of deep tendon reflexes in case 2; this may have been due to the peripheral neuropathy that was clearly evident in nerve conduction studies. T2W and T2W FLAIR images obtained from MRI of the brain also revealed hyperintense lesions involving the corticospinal tracts, optic radiation, and corpus callosum. WES also identified two *GALC* mutations in this patient, one novel and one known. It was notable that MRI of the spinal cord also revealed hyperintensity in the cervical cord on T2W images, thus affecting the spinocerebellar tracts. The myelopathy that was evident in this patient may have contributed to his unsteady gait and dysarthria. The peripheral neuropathy along with pyramidal tract dysfunction also contributed to the gait disturbance. Furthermore, the lesions located in the spinal cord did not affect the corticospinal tracts or spinal cord anterior horn, but may account for the milder weakness in the lower limbs compared to case 1, who needed a wheelchair.

In early publication of the first case of KD, destruction of the medullary sheaths in the spinal cord was described (7). Several subsequent studies of KD, involving histopathology and molecular analysis, described the involvement of the spinal cord in animal models or infants and fetuses with KD. Another study reported reduced levels of GALC enzyme activity in the twitcher mouse (a model of globoid cell leukodystrophy), along with demyelination in spinal cord (8, 9). High levels of psychosine have also been reported in the spinal cord of the fetus and infants with KD (10). Studies involving fetuses with KD reported typical globoid cells in the white matter of the spinal cord and typical intracellular inclusions in cells of the spinal cord, as demonstrated by light and electron microscopy (11, 12). However, published literature very rarely reports myelopathy in patients with KD. We reviewed the existing literature relating to KD patients with myelopathy (Table 1). Thus far, only nine cases, including the cases described in the present study, have reported myelopathy in KD; three of these cases were late infantile onset, one was juvenile onset, and five were adult onset. When examined by MRI, there is a significant diversity in the presentation of myelopathy in patients with KD. MRI of the infantile-onset KD patients with myelopathy showed enhancement of the lower thoracic, lumbar, and lumbosacral nerve roots on T1W postgadolinium enhancement images and thickening of the spinal cord (13–15), while MRI of the late-onset KD patients with myelopathy showed atrophy and intramedullary lesions of the spinal cord on the T2W or T2W FLAIR images (16–19). The cases presented herein, particularly case 1, presented with typical clinical presentations of myelopathy; other previously published cases did not present with such symptoms. Our cases also exhibited intramedullary spinal cord lesions in both the cervical and thoracic cords; these features were also consistent with the clinical symptoms. In spite of the fairly low incidence of late-onset KD, physicians might consider KD as a diagnosis for adult patients presenting with slowly progressive motor dysfunction with typical myelopathy, and the MRI of whom shows hyperintense lesions involving the corticospinal tracts, optic radiation, and corpus callosum on the T2W or T2W FLAIR images. In that case, physicians should implement genetic tests like WES, if possible. Our genetic analysis identified four different *GALC* mutations in our two cases of KD with myelopathy, thus indicating the complex correlation between phenotype and genotype in patients with KD.

**Table 1:** A review of the previous literature relating to KD patients with myelopathy in comparison with the two patients presented in the current report.

**Patient number**	**Gender/Age**	**Origin**	**Age at onset**	**Clinical symptoms**	**MRI of brain**	**MRI of spinal cord**	**Mutations**		**References**
							**Allele 1**	**Allele 2**	
1	Male/ 12 months	United States of America	7 months	Rigidity, opisthotonos posture, irritability, partial seizures	Normal	Diffuse enhancement of the nerve roots of the lower thoracic and lumbar spine from T11 through the cauda equina on T1W postgadolinium images	NA	NA	(13)
2	Male/ 16 months	United States of America	13 months	Motor regression, fever, vomiting and diarrhea, feeding difficulty, irritability, spasticity	Symmetric hyperintensity within the centrum semiovale on T2W and T2W FLAIR images	Enhancement of the lumbosacral nerve roots on T1W postgadolinium images	NA	NA	(14)
3	Female/ 7 years	India	7 months	Excessive crying, feeding difficulty, delayed milestones, spasticity	Hyperintensity in corticospinal tracts, deep periventricular white matter, internal capsule and corpus callosum on T2W images; Generalized cerebral cortical atrophy	Thickening of the cervical cord	NA	NA	(15)
4	Female/ 28 years	Sweden	23 years	Spastic paraparesis.	Hyperintensity along the bilateral corticospinal tracts and in the posterior corpus callosum on T2W FLAIR images	Thinning of the spinal cord	c.1543C>T	c.1543C>T	(16)
5	Female/ 50 years	Japan	30 years	Spastic tetraparesis, amyotrophy, hypoesthesia	Hyperintensity along the bilateral corticospinal tracts on T2W FLAIR images. Enlargement of the bilateral brachial plexus	Atrophy of the cervical spinal cord	NA	NA	(17)#case 2
6	Male/ 20 years	China	20 years	Hemiparesis, Pes Cavus, tendon hyperreflexia, Babinski signs	Hyperintensity along the bilateral corticospinal tracts on T2W and T2W FLAIR images	Hyperintensity in the cervical cord segment of corticospinal tracts on T2W images	p.L634S	p.L634X	(18)
7	Male/ 25 years	China	5 years	Spastic paraparesis, vision loss, nystagmus, Pes Cavus, tendon hyperreflexia, Babinski signs, ankle clonus	Hyperintensity along the bilateral corticospinal tracts on T2W and T2W FLAIR images	Mild atrophy of the spinal cord	p. D46Y	p. G289R	(19)#case 1
8	Male/ 37 years	China	27 years	Tetraparesis, amyotrophy, hypoesthesia, deep sensory impairment, tendon hyperreflexia, Babinski signs, urinary dysfunction, erectile dysfunction, constipation	Hyperintensity along the bilateral corticospinal tracts, and in the optic radiation and corpus callosum on T2W FLAIR images	Hyperintensity in the thoracic cord on T2W images	p.T633Tfs*2	p.T529M	This report
9	Male/ 39 years	China	30 years	Tetraparesis, dysarthria, amyotrophy, fasciculation, hypoesthesia, absent deep tendon reflexes, Babinski signs	Asymmetric hyperintensity along the bilateral corticospinal tracts and in the optic radiation and corpus callosum on T2W and T2W FLAIR images	Hyperintensity in the cervical cord on T2W images	p.P154H	p.W355C	This report

*MRI, magnetic resonance imaging; T1W, T1-weighted; T2W, T2-weighted; T2W FLAIR, T2-weighted fluid-attenuated inversion recovery; NA, not available*.

## Conclusion

In this paper, we reported two Han Chinese males with adult-onset KD with myelopathy. WES identified four *GALC* mutations in these two patients, two known mutations and two novel mutations. Our findings provide a better understanding of the phenotypic and genotypic profiles of adult-onset KD. The complexity and diversity of clinical presentations of KD, including those revealed by MRI, can lead to significant difficulty in terms of diagnosis. Our current findings show that WES is a useful tool with which to support diagnostic decisions. Physicians should consider KD as a diagnosis in cases with progressive motor dysfunction or gait disorder associated with typical myelopathy.

## Data Availability Statement

The original contributions presented in the study are included in the article/Supplementary Material, further inquiries can be directed to the corresponding author/s.

## Ethics Statement

The studies involving human participants were reviewed and approved by The Ethics Committee of Xiangya Hospital, Central South University. The patients/participants provided their written informed consent to participate in this study. Written informed consent was obtained from the individual(s) for the publication of any potentially identifiable images or data included in this article.

## Author Contributions

QX conceived the study. JZ drafted the manuscript. JZ, FJ, and JC participated in the clinical management of patients and data collection. JL, HY, QX, and QZ revised the manuscript. QX and QZ accept responsibility for final approval. All authors contributed to the article and approved the submitted version.

## Conflict of Interest

The authors declare that the research was conducted in the absence of any commercial or financial relationships that could be construed as a potential conflict of interest.
