# Supplementary material for: Novel GALC Mutations Cause Adult-Onset Krabbe Disease With Myelopathy in Two Chinese Families: Case Reports and Literature Review
Source: Front Neurol. 2020 Aug 21;11:830. doi: 10.3389/fneur.2020.00830 (PMC7473299; doi:10.3389/fneur.2020.00830)
Supplement: Supplementary file 1 [file Data_Sheet_1.doc]

Supplementary Material

**Supplementary Table 1.** Nerve conduction studies for Case 1.

| Motor nerve conduction study | | | | | | | |
| --- | --- | --- | --- | --- | --- | --- | --- |
| Nerve | Tract | DML (ms) | | CMAP amplitude (μV) | | MCV (m/s) | |
|  |  | R | L | R | L | R | L |
| Median | Wrist-APB | 3.69 | 3.76 | 14.50 | 14.40 | - | - |
|  | Elbow-wrist | 6.72 | 6.68 | 14.30 | 13.60 | 59.40 | 61.60 |
| Ulnar | Wrist-ADM | 2.44 | 2.25 | 15.40 | 14.50 | - | - |
|  | Elbow-wrist | 5.92 | 6.08 | 14.60 | 14.20 | 60.30 | 57.40 |
| Peroneal | Ankle-EDB | 4.20 | 4.30 | 0.30 | 2.40 | - | - |
|  | Capitula fibula-ankle | 9.34 | 9.43 | 0.29 | 1.79 | 56.40 | 56.50 |
| Tibial | Ankle-AH | 2.61 | 2.62 | 19.90 | 20.40 | - | - |
|  | Politeal fossa-ankle | 9.81 | 9.87 | 15.20 | 17.90 | 45.80 | 45.50 |
| Sensory nerve conduction study | | | | | | | |
| Nerve | Tract | DML (ms) | | SAP  amplitude (μV) | | SCV (m/s) | |
|  |  | R | L | R | L | R | L |
| Median | Digit III-wrist | 2.56 | 2.54 | 42.20 | 42.20 | 54.70 | 59.10 |
| Ulnar | Digit V-wrist | 2.13 | 2.07 | 20.90 | 21.10 | 51.60 | 53.10 |
| Sural | Sura-ankle | 1.83 | 1.80 | 33.30 | 30.20 | 54.60 | 55.60 |

DML: distal motor latency; CMAP: compound muscle action potential; MCV: motor conduction velocity; SAP: sensory action potential; SCV: sensory conduction velocity; APB: abductor pollicis brevis; ADM: abductor digiti minimi; EDB: extensor digitorum brevis; AH: abductor hallucis; -: not available

**Supplementary Table 2.** Nerve conduction studies for Case 2.

| Motor nerve conduction study | | | | | | | |
| --- | --- | --- | --- | --- | --- | --- | --- |
| Nerve | Tract | DML (ms) | | CMAP amplitude (μV) | | MCV (m/s) | |
|  |  | R | L | R | L | R | L |
| Median | Wrist-APB | 6.60 | 0 | 2.20 | 0 | - | - |
|  | Elbow-wrist | 17.80 | 0 | 1.00 | 0 | 20.60 | 0 |
| Ulnar | Wrist-ADM | 5.80 | 0 | 6.4 | 0 | - | - |
|  | Elbow-wrist | 16.80 | 0 | 8.20 | 0 | 24.50 | 0 |
| Peroneal | Ankle-EDB | 0 | 0 | 0 | 0 | - | - |
|  | Capitula fibula-ankle | 0 | 0 | 0 | 0 | 0 | 0 |
| Tibial | Ankle-AH | 0 | 0 | 0 | 0 | - | - |
|  | Politeal fossa-ankle | 0 | 0 | 0 | 0 | 0 | 0 |
| Sensory nerve conduction study | | | | | | | |
| Nerve | Tract | DML (ms) | | SAP  amplitude (μV) | | SCV (m/s) | |
|  |  | R | L | R | L | R | L |
| Median | Digit III-wrist | 0 | 0 | 0 | 0 | 0 | 0 |
| Ulnar | Digit V-wrist | 0 | 0 | 0 | 0 | 0 | 0 |
| Sural | Sura-ankle | 0 | 0 | 0 | 0 | 0 | 0 |

DML: distal motor latency; CMAP: compound muscle action potential; MCV: motor conduction velocity; SAP: sensory action potential; SCV: sensory conduction velocity; APB: abductor pollicis brevis; ADM: abductor digiti minimi; EDB: extensor digitorum brevis; AH: abductor hallucis; -: not available
